# Novel risk loci encompassing genes influencing *STAT3*, GPCR, and oxidative stress signaling are associated with co-morbid GERD and COPD

**DOI:** 10.1371/journal.pgen.1011531

**Published:** 2025-02-07

**Authors:** Ava C. Wilson, Alison Rocco, Joe Chiles, Vinodh Srinivasasainagendra, Wassim Labaki, Deborah Meyers, Bertha Hidalgo, Marguerite R. Irvin, Surya P. Bhatt, Hemant Tiwari, Merry-Lynn McDonald

**Affiliations:** 1 Department of Biostatistics, Harvard T.H. Chan School of Public Health, Harvard University, Boston, Massachusetts, United States of America; 2 Division of Pulmonary, Allergy and Critical Care Medicine, Department of Medicine, University of Alabama at Birmingham, Birmingham, Alabama, United States of America; 3 Department of Biostatistics, School of Public Health, University of Alabama at Birmingham, Birmingham, Alabama, United States of America; 4 Division of Pulmonary and Critical Care Medicine, Michigan Medicine, University of Michigan, Ann Arbor, Michigan, United States of America; 5 Division of Genetics, Genomics, and Precision Medicine, University of Arizona, Tucson, Arizona, United States of America; 6 Department of Epidemiology, School of Public Health, University of Alabama at Birmingham, Birmingham, Alabama, United States of America; 7 Department of Genetics, University of Alabama at Birmingham, Birmingham, Alabama, United States of America; Stanford University, UNITED STATES OF AMERICA

## Abstract

Chronic obstructive pulmonary disease (COPD) is a leading cause of death globally. Gastroesophageal reflux disease (GERD) is a common comorbidity in COPD associated with worse pulmonary symptoms, reduced quality of life, and increased exacerbations and hospitalizations. GERD treatment in COPD is associated with a lower risk of exacerbations and mortality; however, it is not clear whether these findings can be attributed to aging populations where both diseases are likely to co-occur or reflect shared etiology. To test for the influence of common etiology in both diseases, we aimed to identify shared genetic etiology between GERD and COPD. We performed the first whole-genome sequence association analysis of comorbid GERD and COPD in 12,438 multi-ancestry participants. The co-heritability of GERD and COPD was 39.7% (h^2^ = 0.397, SE = 0.074) and we identified several ancestry-independent loci associated with co-morbid GERD and COPD (within *LINC02493* and *FRYL*) known to be involved in oxidative stress and G protein-coupled receptor (GPCR) signaling mechanisms. We found several loci associated with co-morbid GERD and COPD previously associated with GERD or COPD individually, including *HCG17*, which plays a role in oxidative stress mechanisms. Gene set enrichment identified GPCR signaling pathways in co-morbid GERD and COPD loci. Rare variants in *ZFP42*, encoding key regulators of the IL6/*STAT3* pathway, have been previously implicated with GI disorders and were associated with co-morbid GERD and COPD. We identified common genetic etiology for GERD in COPD which begins to provide a mechanistic foundation for the potential therapeutic utility of *STAT3*, oxidation, and GPCR signaling pathway modulators in both GERD and COPD.

## Author summary

GERD is highly prevalent and associated with worse morbidity and mortality in COPD, a leading cause of death worldwide whose prevalence is rising. There is evidence suggesting shared origins of disease between COPD and GERD, which have not been fully explored. Our study represents the largest genomic study of co-morbid GERD and COPD to date, utilizing data from large biobanks to identify risk loci associated with both GERD and COPD. We found that the co-heritability of GERD and COPD was high (39.7%). Further, we identified and confirmed loci associated with co-morbid GERD and COPD influencing *STAT3*, oxidative stress, and G-coupled protein receptor (GPCR) signaling. These results are important because there are currently no drug therapies that stop COPD progression or reduce mortality, however, many medications for GERD are available and easily accessible. Further study of the genes at these loci may enable the prevention of COPD via GERD inhibition.

## Introduction

Chronic Obstructive Pulmonary Disease (COPD) is a leading cause of death worldwide [1]. COPD is a chronic, progressive illness characterized by increased lung inflammation and airflow limitation that is not fully reversible [2]. COPD is one of the few chronic diseases whose prevalence continues to rise [3]. In the United States, COPD contributes to a high economic burden with direct costs estimated to be $32 billion per year and indirect costs accounting for an additional $20 billion annually [4]. Compared to healthy, aged-matched adults, COPD patients average a higher number of comorbidities impacting survival, quality of life, and healthcare expenditures [5,6]. Despite the enormous burden of COPD, there are currently no pharmacologic therapies that stop disease progression or reduce mortality, leaving a huge unmet need for therapies. Gastroesophageal reflux disease (GERD) is a highly prevalent comorbidity in COPD, ranging in prevalence from 19% to 78%, with an average of 24% [7,8]. GERD has pharmacologic treatments however, its complex relationship with COPD is poorly understood [9].

GERD symptoms are more common in COPD patients compared to controls without COPD [10]. GERD can exacerbate COPD and *vice versa*, and mechanical changes in COPD can aggravate GERD [8]. Reflux theory postulates that micro-aspiration of gastric contents damages the lungs as a direct mechanism of how GERD can influence the development of COPD [8]. In COPD, mechanical changes, increased cough frequency, and COPD medications all can aggravate reflux, contributing to GERD [8]. COPD medications, specifically beta-agonists, corticosteroids, anticholinergics, and theophylline have been shown to alter both esophageal tone (reduced lower esophageal sphincter pressure and/or esophageal motility) and respiratory mechanics (increased lung hyperinflation) [8]. These mechanisms can instigate a compromised antireflux barrier, leading to increased GERD severity or development [8].

COPD and GERD are highly heritable and there is evidence supporting the role of shared etiology in COPD and GERD. Specifically, heritability estimates of COPD have ranged from 35% to 43% [11–14], whereas, heritability estimates of GERD have ranged from 31% to 43% [15–17]. However, the shared heritability of COPD and GERD has not been investigated. Nonetheless, genetic variants in the *CSMD1* and *MAD1L1* genes have been previously associated with GERD as well as with COPD providing support for the role of common etiology between the traits [18–21]. *MAD1L1* is widely expressed in all tissues and is a member of the mitotic spindle-assembly checkpoint, where several mitogenic G-protein-coupled receptors (GPCRs) in the protease-activated receptor family (PAR1-3) relevant to GERD and COPD pathophysiology are also produced [8,22–26]. In COPD, GPCR expression, including the expression of proteinase-activated receptors, leads to inflammation and can be stimulated by gastric acid [25,27–29]. Previous studies have identified intracellular signaling pathways that regulate mitogenic interactions of GPCR agonists with growth factors in airway smooth muscle [25]. These intracellular signaling pathways include the *MAD1L1* gene, as well as genes that encode numerous class A GPCRs including proteinase-activated receptors, as well as opioid, dopamine, acetylcholine muscarinic, and chemokine receptors, among others, implicated in both GERD and COPD pathogenesis [30–36]. Overall, there is evidence suggesting a shared etiology between COPD and GERD, which has not been fully explored. Currently, a major gap remains as there have been no studies attempting to disentangle and elucidate the relationship between comorbid GERD and COPD using genomics.

For this reason, we aimed to investigate the genetic etiology of comorbid GERD and COPD utilizing whole-genome sequencing data from three large cohorts of well-characterized participants with GERD and COPD from the Trans-Omics for Precision Medicine (TOPMed) program (COPDGene, ECLIPSE, and SPIROMICS) [37].

## Results

### Sample demographics

Participants were more likely to be male, apart from SPIROMICS AA (49.1% male, S1 Table). On average, participants from COPDGene tended to be younger (Mean±SD = 59.8±9.1 years) when compared to participants from ECLIPSE (Mean±SD = 62.6±7.7 years) and SPIROMICS (Mean±SD = 63.1±9.0 years) (S1 Table). The frequency of COPD was highest among NHW participants (ranging from 50.5% in COPDGene to 66.2% in ECLIPSE) compared to AA participants where the frequency of COPD was 36.4% in COPDGene and 50.4% in SPIROMICS (S1 Table). The frequency of GERD was highest among NHW from COPDGene (29.4%, S1 Table), compared to SPIROMICS, where the frequency of GERD was highest among AA participants (23.3%, S1 Table). The frequency of comorbid GERD and COPD was highest among NHW participants (ranging from 18.4% in COPDGene to 32.3% in SPIROMICS) in contrast to AA participants (7.0% in COPDGene and 13.3% in SPIROMICS) (S1 Table). On average, AA participants from COPDGene and SPIROMICS were younger and had fewer pack years of smoking, which may account for the lower frequency of COPD and co-morbid GERD and COPD among these participants (S1 Table).

### Heritability of GERD and COPD

Among NHW participants, the co-heritability of GERD and COPD was estimated to be 39.7% (h^2^ = 0.397, 95% CI = 32.2–47.0, Table 1). The heritability of COPD alone was estimated to be 28.3% (h^2^ = 0.283, 95% CI = 23.5–33.1, Table 1) in the combined NHW participants, 35.3% (h^2^ = 0.353, 95% CI = 27.4–43.2, Table 1) in NHW from COPDGene, and 66.2% (h^2^ = 0.662, 95% CI = 54.3–78.0, Table 1) in the combined AA participants. The heritability of GERD alone was estimated to be 0% (h^2^ = -0.01, 95% CI = -0.08–0.06, Table 1) in the combined NHW participants, however, this estimate was not significant. The heritability of GERD, regardless of COPD status, was estimated to be 30.7% (h^2^ = 0.307, 95% CI = 26.9–34.5, Table 1) in the combined NHW participants.

**Table 1:** Narrow sense heritability (h^2^) estimates for co-morbid GERD and COPD and COPD only using WGS data. h^2^ was estimated from participants from TOPMed cohorts (COPDGene, ECLIPSE, SPIROMICS).

**GERD + COPD**			
**Cohort**	**N**	**h**^**2**^ **LDAK**	**95% CI LDAK**
Total NHW	4,402	39.7	32.2–47.0
**COPD Only**			
**Cohort**	**N**	**h**^**2**^ **LDAK**	**95% CI LDAK**
Total NHW	6,921	28.3	23.5–33.1
COPDGene NHW	4,158	35.3	27.4–43.2
Total AA	2,630	66.2	54.3–78.0
**GERD Only**			
**Cohort**	**N**	**h**^**2**^ **LDAK**	**95% CI LDAK**
Total NHW^1^	3,296	-0.01	-0.08–0.06
Total NHW^2^	9,052	30.7	26.9–34.5

1. Cases consisted of GERD only, excluding participants with COPD.

2. Cases consisted of GERD, regardless of COPD status.

AA–African American; CI - Confidence Interval; COPD–Chronic Obstructive Pulmonary Disease; GERD–Gastroesophageal Reflux Disease; NHW–Non-Hispanic White; WGS–Whole Genome Sequencing.

### Findings from WGS Analyses with Co-Morbid GERD and COPD, COPD Only, and GERD Only

First, we summarize comorbid GERD and COPD findings for the multi-ancestry, NHW, and AA participants starting with single variant results followed by, aggregate association testing of rare (MAF<5%) variants and GSEA. Then, we summarize our findings from COPD only analyses followed by GERD only analyses for the multi-ancestry, combined NHW, and combined AA participants starting with single variant results followed by aggregate association testing and GSEA (S1 Text).

### Single SNVs Associated with Co-Morbid GERD and COPD

**Fig 1:**
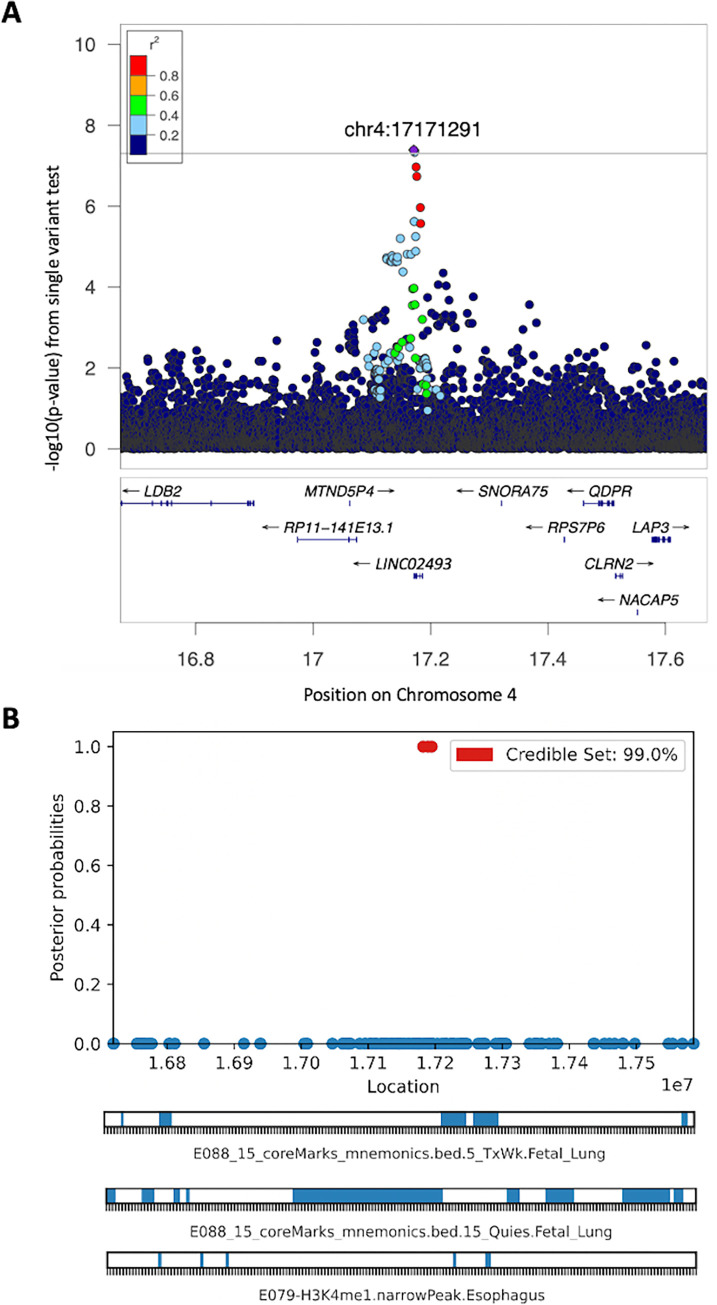
Fine-mapping of the *LINC02493* region associated with co-morbid GERD and COPD in multi-ancestry GWAS analysis. (**A**) Regional association plot and (**B**) functional annotation tracks. H3K4me1 –Monomethylation of lysine 4 on histone H3 protein subunit; TxWk–weak transcription.

No loci passed genome-wide significance for association with co-morbid GERD and COPD. Seventy-four independent loci were associated (P<5E-06) with co-morbid GERD and COPD across all multi-ancestry, combined NHW, and combined AA participants (S2 Table). In multi-ancestry analyses, 34 independent loci were associated with co-morbid GERD and COPD (Tables 2 and S3). Of these, 4:17172652 (intronic to *LINC02493*), was the top variant associated with co-morbid GERD and COPD [OR = 1.55, 95% CI = 1.42–1.68, P = 1.92E-07, respectively, S3 Table and Fig 1]. One variant (14:52006654) within the exonic region of *NID2* was suggestively associated with co-morbid GERD and COPD [OR = 10.26, 95% CI = 6.18–17.06, P = 3.84E-06, S3 Table]. Among multi-ancestry participants, we also identified 4 ancestry independent loci suggestively associated (P<5E-6) with co-morbid GERD and COPD whose representative genes were *LINC02493*, *FRYL*, (*LOC643542 –TMX3)*, and (*XPO1 –FAM161A*) (S2 Table). In the combined NHW participants, 35 variants from 18 independent loci were suggestively associated with co-morbid GERD and COPD (S2 and S4 Tables). The top variant associated with comorbid GERD and COPD in the combined NHW participants was 4:116174264 [OR = 1.59, 95% CI = 1.45–1.74, P = 3.24E-07, S4 Table], which is intergenic to *NDST4* and *MIR1973*. In the combined AA participants, 107 variants from 22 independent loci were associated with co-morbid GERD and COPD (S2 and S5 Tables). One variant (7:14108277), intergenic to *ETV1* and *DGKB*, was significantly associated with comorbid GERD and COPD in the single variant analysis [OR = 1.86, 95% CI = 1.66–2.08, P = 3.08E-08, S5 Table] and in meta-analysis [OR = 1.89, 95% CI = 1.67–2.08, P = 2.47E-08, S5 Table] at levels withstanding GWS.

**Table 2:** Independent significant loci (P<5E-06) associated with comorbid GERD and COPD in multi-ancestry participants from COPDGene, ECLIPSE, and SPIROMICS.

Locus	Chr	Start (bp)	Stop (bp)	Gene(s) within Locus	Lead SNV	MAF	OR	95% CI LL	95% CI UL	P
GC1	4	17125315	17192549	LINC02493*	4:17172652	0.0701	1.55	1.42	1.68	1.92E-07
GC2	15	99747875	99747875	*LYSMD4*,*DNM1P46*	15:99747875	0.0147	0.42	0.36	0.5	3.60E-07
GC3	4	7542883	7567991	*SORCS2*	4:7542883	0.0936	1.48	1.37	1.6	4.66E-07
GC4	4	47834938	48967098	***FRYL*** ^***±***^	4:48740358	0.0033	15.82	9.71	25.76	5.23E-07
GC5	6	28658430	29561292	*LOC101929006*,*OR14J1*	6:29299136	0.0121	3.51	2.76	4.46	6.09E-07
GC6	3	151027254	151027254	*CLRN1-AS1*	3:151027254	0.3861	0.79	0.76	0.83	6.27E-07
GC7	18	67928217	68040033	***LOC643542*,*TMX3*** ^***±***^	18:67956064	0.0252	2.41	2.02	2.87	1.34E-06
GC8	4	144341775	144585304	*GYPA*,*HHIP-AS1*	4:144513592	0.3291	0.8	0.76	0.84	1.44E-06
GC9	4	116125069	116254463	*RLFP1*, TTC39CP1*	4:116174264	0.0586	1.52	1.39	1.67	2.03E-06
GC10	15	97502536	97506289	***LINC02254*** ^***+***^	15:97503125	0.0096	4.08	3.08	5.39	2.14E-06
GC11	11	8380420	8407223	*STK33*	11:8396074	0.0153	0.34	0.27	0.42	2.33E-06
GC12	20	13256158	13256158	***ISM1*** ^***+***^	20:13256158	0.0238	2.5	2.07	30.1	2.37E-06
GC13	6	47312126	47341972	*TNFRSF21*,*CD2AP*	6:47318522	0.0042	8.82	5.78	13.45	2.56E-06
GC14	18	71156136	71156136	*GTSCR1*,*LINC01541*	18:71156136	0.0075	2.97	2.35	3.76	2.94E-06
GC15	5	18315039	18315039	*LINC02223*,*CDH18*	5:18315039	0.0026	8.72	5.56	13.67	2.97E-06
GC16	16	5604604	5604604	*LINC01570*	16:5604604	0.0188	2.62	2.14	3.19	3.01E-06
GC17	19	13600835	13601811	*CACNA1A*,*CCDC130*	19:13601811	0.3367	1.25	1.19	1.3	3.03E-06
GC18	2	179390116	179440687	*SESTD1*,*ZNF385B*	2:179390116	0.0111	0.4	0.33	0.49	3.03E-06
GC19	2	103160979	103638750	*LINC01935*,*LOC100287010*	2:103220143	0.0114	3.63	2.79	4.72	3.05E-06
GC20	11	76671438	76683811	*LRRC32*	11:76671438	0.005	0.27	0.2	0.35	3.25E-06
GC21	2	61537939	61616048	*XPO1*,FAM161A*	2:61597797	0.3451	0.81	0.77	0.84	3.39E-06
GC22	2	10097737	10097737	*CYS1*,*RRM2*	2:10097737	0.0089	0.37	0.29	0.45	3.49E-06
GC23	11	120101226	120167154	*LOC102724301*,*TRIM29*	11:120110021	0.1648	1.31	1.24	1.39	3.51E-06
GC24	14	52006654	52006654	*NID2*	14:52006654	0.0016	10.26	6.18	17.06	3.84E-06
GC25	9	81912216	82486579	*LOC101927502*,*SPATA31D5P*	9:81912216	0.0034	0.19	0.13	0.28	3.98E-06
GC26	11	109622352	109760703	*C11orf87*,*ZC3H12C*	11:109629338	0.0032	11.33	7.05	18.2	4.07E-06
GC27	21	42786584	42879574	*LINC01668*,*WDR4*	21:42786584	0.0287	0.56	0.5	0.64	4.33E-06
GC28	6	155448889	155877188	*LOC105378068*,*MIR1202*	6:155556053	0.0025	0.16	0.1	0.24	4.38E-06
GC29	2	214760493	214812934	*BARD1*	2:214792971	0.1117	1.4	1.3	1.51	4.40E-06
GC30	4	189131080	189131080	*LINC01060*,*LINC01262*	4:189131080	0.005	4.01	2.95	5.44	4.41E-06
GC31	8	2180140	2180140	*MYOM2*,*LOC101927815*	8:2180140	0.0077	2.88	2.28	3.63	4.43E-06
GC32	10	8371381	8372980	*LINC00708*,*LOC105376398*	10:8371902	0.0079	4.99	3.61	6.9	4.53E-06
GC33	2	216980487	216980487	***LINC01921*,*DIRC3-AS1*** ^***+***^	2:216980487	0.0085	4.53	3.33	6.16	4.68E-06
GC34	11	3914824	4403491	*TRIM21*,*OR52K2*	11:4403491	0.0018	9.5	5.78	15.62	4.86E-06

Bold*—denotes variant is present in analyses of combined NHW populations.

**Bold**^**+**^**—**denotes variant is present in analyses of combined AA populations.

**Underline–**denotes smaller p-value in multi-ancestry analyses compared to ancestry stratified analyses.

Abbreviations: BP–Base Pair; Chr–Chromosome; CI–Confidence Interval; COPD–Chronic Obstructive Pulmonary Disease; GERD–Gastroesophageal Reflux Disease; LL–Lower Limit MAF–Minor Allele Frequency; OR–Odds Ratio; SNV–Single Nucleotide Variant; UL–Upper Limit.

### Gene-based Regions of Rare Variants Associated with GERD and COPD

Among multi-ancestry participants, gene-based analysis of rare variants indicated *SLC16A8* was the top gene associated with co-morbid GERD and COPD, however, it was not genome-wide significant (transcript ENST00000427592, P = 4.77E-05, S6 Table). Among NHW participants, rare variants within *ZFP42* mapping to three transcripts (ENST00000326866, P = 1.3E-05; ENST00000509524, P = 1.3E-05; ENST00000618147, P = 1.3E-05; S6 Table) were the top regions associated with co-morbid GERD and COPD. Among AA participants, rare variants within the *KRTAP3-1* gene were associated with co-morbid GERD and COPD, although they were not genome-wide significant (P = 1.94E-04, S6 Table).

### Gene-set Enrichment Analyses of Genes Within Co-Morbid GERD and COPD Associated Loci

To gain insight into the overall etiological role of genetic variation within comorbid GERD and COPD, we examined enrichment of genes within loci associated with comorbid GERD and COPD from single variant analyses. Genes mapping to loci associated with COPD and GERD among multi-ancestry participants were enriched with genes involved in asthma and major depressive disorder (q-value = 3.26E-18, S7 Table), GPCR signaling (WP GPCRs Class A Rhodopsinlike, q-value = 0.0014, S7 Table) and lung cancer (Lung cancer in ever smokers, q-value = 2.27E-05, S7 Table), among other known-gene sets. Genes mapping to loci associated with COPD and GERD among NHW participants showed enrichment of genes involved in erectile dysfunction (Erectile dysfunction, q-value = 9.06E-09, S7 Table), among others. No gene set within the combined AA participants withstood correction for multiple comparisons.

### Comparison of COPD and GERD Genetic Architecture to COPD Only and GERD Only

**Fig 2:**
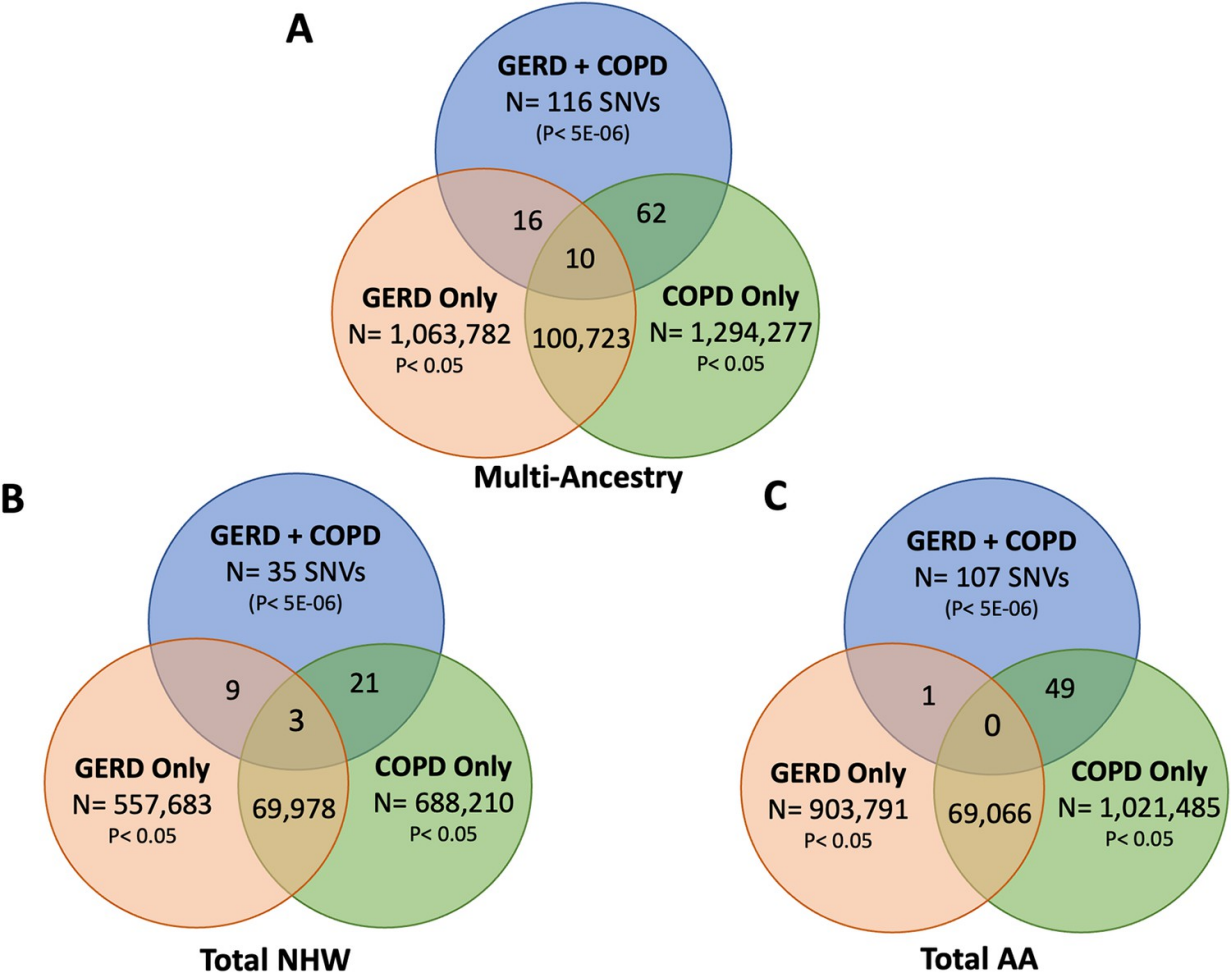
Venn diagrams depicting the overlap between SNVs suggestively associated (P< 5E-6) with co-morbid GERD and COPD and SNVs with a P-value < 0.05 associated with GERD only and COPD only from multi-ancestry (A), total NHW (B), and total AA (C) GWAS analyses. AA–African American; COPD–Chronic Obstructive Pulmonary Disease; GERD–Gastroesophageal Reflux Disease; GWAS–Genome-wide Association Study; NHW–Non-Hispanic White; SNV–Single Nucleotide Variant.

The overlap in variants between the combined COPD and GERD, COPD only, and GERD only GWAS analyses for multi-ancestry, combined NHW, and combined AA participants are summarized in Tables 3 and 4. In the multi-ancestry population, 10 variants with a p< 0.05 in both the COPD only and GERD only analyses overlapped with the variants suggestively associated with co-morbid GERD and COPD (Fig 2 and S3 Table). Of these variants, one variant was intergenic to *LOC102724301* and *TRIM29*, one variant was intergenic to *CACNA1A* and *CCDC130*, and 8 variants were located within the intronic region of *FRYL* (S3 Table). In the combined NHW population, 3 variants with a p< 0.05 in both the COPD only and GERD only analyses overlapped with the variants suggestively associated with co-morbid GERD and COPD (Fig 2 and S4 Table). One variant was intergenic to *SAGE1* and *MMGT1*, one variant was located within the 3’-untranslated region of *SAGE1*, and one variant was intergenic to *RAB3C* and *PDE4D* (S4 Table). No variants within the combined AA COPD only and GERD only GWAS analyses with a significance threshold of P< 0.05 overlapped with variants suggestively associated with co-morbid GERD and COPD.

**Table 3:** Independent significant loci (P<5E-06) associated with COPD in multi-ancestry participants from COPDGene, ECLIPSE, and SPIROMICS.

Locus	Chr	Start (bp)	Stop (bp)	Gene(s) within Locus	Lead SNV	MAF	OR	95% CI LL	95% CI UL	P-Value
C1	4	88838604	89103053	FAM13A*	4:88945562	0.4482	1.22	1.18	1.26	6.83E-09
C2	15	78455574	78623530	*CHRNA3*	15:78606381	0.3601	1.23	1.19	1.28	1.07E-08
C3	4	144341775	144587571	*GYPA*,HHIP-AS1*	4:144513749	0.3244	0.82	0.79	0.85	5.99E-08
C4	1	97504891	97895884	***DPYD*** ^***±***^	1:97699684	0.0017	6.96	4.79	10.11	7.40E-08
C5	14	92603635	92665338	RIN3*	14:92631994	0.1314	0.76	0.72	0.8	1.01E-07
C6	1	208228256	208228256	***PLXNA2*** ^***±***^	1:208228256	0.0064	2.84	2.33	3.47	1.47E-07
C7	15	71301584	71336031	***THSD4*** ^***±***^	15:71317183	0.3428	1.2	1.16	1.25	2.34E-07
C8	3	127996353	128273095	EEFSEC*	3:128273095	0.0869	0.74	0.69	0.78	3.63E-07
C9	16	1668109	1683893	*CRAMP1*	16:1668109	0.0104	0.44	0.37	0.52	3.71E-07
C10	6	71077348	71120744	*B3GAT2*,OGFRL1*	6:71077348	0.0017	0.13	0.09	0.2	4.36E-07
C11	2	61186588	61632113	*XPO1*,*FAM161A*	2:61632113	0.2117	1.24	1.19	1.29	5.68E-07
C12	15	98973753	98973753	***PGPEP1L*** ^***±***^	15:98973753	0.0051	3.04	2.43	3.8	7.00E-07
C13	12	30087497	30115004	*TMTC1*,*IPO8*	12:30092962	0.0057	3.05	2.43	3.81	7.63E-07
C14	11	44597554	45712842	***TSPAN18*** ^***±***^	11:44896643	0.0013	8.17	5.28	12.64	7.74E-07
C15	6	125868155	125868155	***NCOA7*** ^***±***^	6:125868155	0.012	2.11	1.82	2.46	8.34E-07
C16	10	110727534	110797284	RBM20*	10:110737301	0.0035	0.25	0.19	0.33	9.31E-07
C17	6	71947154	72598492	*RIMS1*,KCNQ5*	6:72598492	0.0016	0.12	0.08	0.19	1.11E-06
C18	16	28498723	28532060	*APOBR*,IL27*	16:28499072	0.45	1.18	1.14	1.22	1.15E-06
C19	10	115981583	116055822	***ATRNL1*,*GFRA1*** ^***±***^	10:115985709	0.012	2.09	1.79	2.43	1.27E-06
C20	12	38028316	38610176	*NONE*,ALG10B*	12:38184110	0.0063	0.36	0.29	0.45	1.29E-06
C21	8	6727486	8061074	*DEFA5*,*LINC00965*	8:7092908	0.0016	7.22	4.78	10.91	1.58E-06
C22	9	3768845	3779664	***RFX3-AS1*,*GLIS3*** ^***+***^	9:3768845	0.0082	2.39	1.99	2.86	1.66E-06
C23	4	156966205	156966707	PDGFC*	4:156966205	0.2804	0.83	0.8	0.86	1.67E-06
C24	12	81074860	81144532	***ACSS3*** ^***+***^	12:81129256	0.0968	1.31	1.24	1.38	1.79E-06
C25	20	610570	610570	***TCF15*** ^***±***^	20:610570	0.0067	2.55	2.1	3.11	1.89E-06
C26	1	48218610	48218610	***SKINT1L*,*SLC5A9*** ^***+***^	1:48218610	0.0012	9.29	5.84	14.78	1.93E-06
C27	9	14074023	14140920	*NFIB*	9:14100066	0.4803	1.19	1.14	1.23	1.94E-06
C28	8	70120938	70962590	*NCOA2*,LOC101926892*	8:70429086	0.003	0.23	0.17	0.32	2.13E-06
C29	9	73222279	73584645	***ANXA1*,*LOC101927358*** ^***±***^	9:73524131	0.0156	1.89	1.65	2.16	2.23E-06
C30	8	123252687	123367515	*ZHX1*,*ZHX1-C8orf76*	8:123252687	0.2149	0.82	0.79	0.86	2.68E-06
C31	2	80624982	80630975	*CTNNA2*	2:80630834	0.1063	0.77	0.73	0.81	2.78E-06
C32	8	87195565	88024136	*DCAF4L2*,*MMP16*	8:88024136	0.0099	2.25	1.89	2.68	2.84E-06
C33	17	77369348	77369348	SEPT9*	17:77369348	0.0024	0.19	0.13	0.27	2.88E-06
C34	17	1931941	1943130	***RPA1*,*RTN4RL1*** ^***+***^	17:1931941	0.0029	4.3	3.15	5.86	2.99E-06
C35	1	17704770	17708993	*ARHGEF10L*,*ACTL8*	1:17708429	0.0997	0.77	0.73	0.81	3.01E-06
C36	14	20817996	20820412	***RNASE1*,*RNASE3*** ^***+***^	14:20818549	0.0147	1.88	1.64	2.16	3.04E-06
C37	6	20779535	20863918	CDKAL1*	6:20779535	0.0195	0.56	0.5	0.64	3.09E-06
C38	21	35714739	35720595	*MIR802*	21:35720595	0.0019	0.17	0.11	0.24	3.43E-06
C39	13	60994461	61423821	*LINC00378*,*MIR3169*	13:60994461	0.0038	3.61	2.74	4.76	3.67E-06
C40	13	73874380	73881789	***KLF12*** ^***+***^	13:73881789	0.0056	2.72	2.19	3.39	3.70E-06
C41	3	7821968	7829252	*GRM7*,*LOC101927394*	3:7821968	0.0041	3.24	2.51	4.18	3.72E-06
C42	10	2545404	2594880	***LOC105376351*,*PFKP*** ^***±***^	10:2594816	0.943	1.39	1.3	1.5	3.89E-06
C43	6	6733307	6751826	*LY86*,*RREB1*	6:6742316	0.3435	1.18	1.14	1.22	3.99E-06
C44	6	42940690	42947011	CNPY3-GNMT*	6:42940690	0.2218	0.83	0.8	0.86	4.01E-06
C45	8	20633755	20745944	*SNORD3F*,*LOC102467222*	8:20653070	0.0172	0.56	0.49	0.64	4.19E-06
C46	2	77212574	77280971	*LRRTM4*	2:77212574	0.0084	2.32	1.93	2.79	4.32E-06
C47	12	68537523	68561557	*LINC02384*,*RAP1B*	12:68560123	0.2936	0.85	0.81	0.88	4.53E-06
C48	22	26331907	26331907	*SEZ6L*	22:26331907	0.0389	0.67	0.61	0.73	4.57E-06
C49	17	37542996	37618045	*SYNRG*	17:37581434	0.0066	2.53	2.07	3.1	4.60E-06
C50	13	54292766	54336605	*MIR1297*,*MIR5007*	13:54336605	0.0029	4.05	2.97	5.52	4.63E-06
C51	8	116465834	116560300	*LINC00536*,EIF3H*	8:116492903	0.1842	0.82	0.79	0.85	4.63E-06
C52	8	10218059	10229274	*MSRA*	8:10218894	0.0085	2.26	1.89	2.71	4.69E-06
C53	10	89571547	89628487	*PANK1*	10:89618323	0.0155	1.85	1.62	2.12	4.87E-06
C54	2	99224578	101024883	*GYPA*,*HHIP-AS1*	2:100180501	0.237	0.83	0.8	0.86	4.99E-06

Bold*—denotes variant is present in analyses of combined NHW populations.

**Bold**^**+**^**—**denotes variant is present in analyses of combined AA populations.

**Underline–**denotes smaller p-value in multi-ancestry analyses compared to ancestry stratified analyses.

Abbreviations: BP–Base Pair; Chr–Chromosome; CI–Confidence Interval; COPD–Chronic Obstructive Pulmonary Disease; LL–Lower Limit MAF–Minor Allele Frequency; OR–Odds Ratio; SNV–Single Nucleotide Variant; UL–Upper Limit.

**Table 4:** Independent significant loci (P<5E-06) associated with GERD in multi-ancestry participants from COPDGene, ECLIPSE, and SPIROMICS.

Locus	Chr	Start (bp)	Stop (bp)	Gene within Locus	Lead SNV	MAF	OR	95% CI LL	95% CI UL	P
G1	12	108633196	108633196	*SELPLG*	12:108633196	0.0303	2.17	1.88	2.49	7.77E-08
G2	13	81925105	82006238	PTMAP5*	13:82006238	0.016	2.64	2.2	3.17	2.36E-07
G3	7	11260345	11260345	*NONE*,*THSD7A*	7:11260345	0.0741	1.58	1.44	1.72	2.72E-07
G4	2	177073198	177136257	*LINC01117*,*HNRNPA3*	2:177126890	0.8986	0.68	0.62	0.73	4.20E-07
G5	2	35558557	35626340	*MIR548AD*,*LOC100288911*	2:35626340	0.5457	1.28	1.21	1.34	7.50E-07
G6	10	3351068	3669843	*LOC105376360*	10:3669371	0.2613	1.31	1.24	1.38	9.87E-07
G7	14	26938397	26938397	ENSG00000258081*	14:26938397	0.1034	1.46	1.35	1.57	1.00E-06
G8	9	134742137	134757805	**COL5A1** ^ **+** ^	9:134755098	0.003	20.99	12.35	35.67	1.05E-06
G9	9	122651147	122665528	*OR1L1*,*OR1L3*	9:122665528	0.014	2.57	2.13	3.12	1.32E-06
G10	13	21298633	21326315	***LINC00539*,*MIPEPP3*** ^***+***^	13:21305520	0.0027	24.16	13.82	42.22	1.32E-06
G11	13	80760666	80760666	*SPRY2*,*LINC00377*	13:80760666	0.0031	7.60	5.07	11.41	1.83E-06
G12	11	85933350	86131738	*CCDC83*,*PICALM*	11:85942182	0.0134	3.61	2.81	4.64	1.86E-06
G13	7	87957472	88917077	*ADAM22*	7:87957472	0.0032	7.58	5.07	11.34	1.95E-06
G14	21	15709380	15731922	*NRIP1*,*USP25*	21:15709380	0.0019	13.37	7.98	22.39	1.96E-06
G15	12	22363424	22405859	***ST8SIA1*,*C2CD5*** ^***±***^	12:22363424	0.0055	7.60	5.18	11.16	2.12E-06
G16	7	126256693	126429783	***LOC101928283*,*GRM8*** ^***+***^	7:126256693	0.0025	21.16	12.24	36.57	2.18E-06
G17	11	107623045	107687501	***ELMOD1*** ^***±***^	11:107655256	0.0274	2.33	1.97	2.77	2.23E-06
G18	1	99730634	99730634	*FRRS1*	1:99730634	0.0144	3.02	2.41	3.78	2.45E-06
G19	19	5755199	5755199	*CATSPERD*	19:5755199	0.011	2.88	2.31	3.59	2.49E-06
G20	17	28018906	28018906	*LINC01992*,*NLK*	17:28018906	0.0182	2.89	2.34	3.58	2.57E-06
G21	2	111633064	111655353	*MIR4435-2HG*,*ANAPC1*	2:111633064	0.0044	5.43	3.85	7.65	2.71E-06
G22	18	10591461	10593208	***NAPG*,*LINC01887*** ^***+***^	18:10593208	0.0021	44	22.54	85.91	3.51E-06
G23	7	25660796	25774071	***LINC03007*** ^***+***^	7:25748396	0.0145	3.32	2.60	4.24	3.69E-06
G24	14	64684035	64817556	*PLEKHG3*	14:64712911	0.0026	8.71	5.58	13.58	3.70E-06
G25	6	33760978	33801120	*IP6K3*,*LEMD2*	6:33768518	0.4406	1.26	1.2	1.32	3.71E-06
G26	5	58127195	59956159	***PDE4D*** ^***+***^	5:59112184	0.0022	28.66	15.5	52.98	3.79E-06
G27	15	93555577	93565472	*RGMA*,*LINC02207*	15:93563203	0.0593	1.78	1.58	2.02	3.91E-06
G28	9	11034423	11641412	***PTPRD-AS2*,*TYRP1*** ^***+***^	9:11034423	0.0031	14.88	8.94	24.75	3.99E-06
G29	8	397694	545416	*FBXO25*,*TDRP*	8:484010	0.0036	10.89	6.88	17.26	4.12E-06
G30	6	35924122	36019348	***SLC26A8*** ^***+***^	6:35943272	0.0037	10.92	6.9	17.3	4.19E-06
G31	11	121383209	121383209	*SC5D*,*SORL1*	11:121383209	0.0036	6.40	4.36	9.39	4.22E-06
G32	13	108711073	108712339	*MYO16*	13:108711073	0.4576	1.26	1.20	1.33	4.42E-06
G33	6	7169509	7183701	*RREB1*	6:7181998	0.0176	2.87	2.31	3.58	4.55E-06

Bold*—denotes variant is present in analyses of combined NHW populations.

**Bold**^**+**^**—**denotes variant is present in analyses of combined AA populations.

**Underline–**denotes smaller p-value in multi-ancestry analyses compared to ancestry stratified analyses.

Abbreviations: BP–Base Pair; Chr–Chromosome; CI–Confidence Interval; GERD–Gastroesophageal Reflux Disease; LL–Lower Limit MAF–Minor Allele Frequency; OR–Odds Ratio; SNV–Single Nucleotide Variant; UL–Upper Limit.

## Discussion

This is the first genomic analysis that begins to disentangle the shared genetic etiology of co-morbid GERD and COPD in 12,438 participants from COPDGene, ECLIPSE, and SPIROMICS with WGS data. Observational studies have provided evidence suggesting an association between GERD and COPD, despite the lack of understanding of a specific cause and effect relationship between these two chronic diseases [7,38]. By leveraging the power of a large-scale GWAS approach, we identified common genetic etiology for GERD in COPD, which supports the epidemiological link between GERD and COPD and begins to provide a mechanistic foundation for the therapeutic utility of the *STAT3*, GPCR, and oxidative stress signaling pathways as modulators in both GERD and COPD.

The narrow sense heritability of co-morbid GERD and COPD was high, 39.7%, in participants of NHW descent. These results are in keeping with previous studies of the heritability of COPD and GERD individually, which range from 35% to 43% for COPD and 31% to 43% for GERD [11–17,39]. The high co-heritability between GERD and COPD may be explained by biological pleiotropic effects on both disorders which our single variant and gene-based analyses support. Interestingly, when participants with COPD were excluded from the heritability estimation of GERD, the heritability was not significant. However, when participants with COPD and GERD were included, the heritability of GERD was 31%. It is likely removing participants with COPD reduced the power necessary to detect GERD heritability. Additionally, this may indicate much of the heritability of GERD is due to pleiotropic variants also associated with COPD. Further, gene-based analysis of rare variants identified three regions within the *ZFP42* (*REX1*) gene, which were associated with co-morbid GERD and COPD. *ZFP42* encodes a key regulator involved in IL6/*STAT3* pathway activation, which has been shown to be triggered by GERD [40,41]. Previous mechanistic studies have shown IL6/*STAT3* pathway activation promotes COPD pathogenesis by increasing neutrophil activity and inflammation, as well as through excessive apoptosis [42]. Increased *ZFP42* expression has been shown to activate the IL6/*STAT3* pathway, which could be further aggravated in the presence of GERD, leading to expedited COPD progression [43].

Further, we identified variants and regions significantly associated with co-morbid GERD and COPD involved in GPCR signaling. The association between GPCR signaling and COPD and GERD progression, individually, has long been recognized, and in fact, drugs targeting GPCR signaling pathway receptors, such as muscarinic acetylcholine and protease-activated receptors, are sought after for both conditions [44–48]. In multi-ancestry participants, we identified 29 variants within one ancestry-independent locus associated with co-morbid GERD and COPD. Further, we identified 8 variants within *FRYL* associated with both COPD only and GERD only that were suggestively associated with co-morbid GERD and COPD, providing further evidence that these variants may be the driver of the co-morbid GERD and COPD phenotype. These variants are intronic to *FRYL*, which encodes a protein involved in actin cytoskeleton regulation [49]. Previous studies have found that actin cytoskeleton dysfunction in smooth muscle is linked to dysfunctional acetylcholine muscarinic receptor (a member of the class A GPCR family) signaling, which induces differential actin cytoskeleton rearrangements [50,51]. Cigarette smoke is associated with increased actin polymer levels, which contributes to the destabilized epithelial cell adhesion seen in COPD pathology [52]. Like COPD, smoking is a risk factor for GERD, and in GERD, acid exposure is associated with increased actin expression in epithelial cells, which could contribute to increased cell adhesion destabilization, further exacerbating COPD pathogenesis [53]. This finding was further supported by GSEA in which we had significant overlap between the top loci associated with co-morbid GERD and COPD and class A GPCR signaling. In the combined AA participants, one intergenic variant (rs7800452) was significantly associated with comorbid GERD and COPD and 36 kb from its nearest gene, *DGKB*. *DGKB*, which encodes diacylglycerol kinase beta, inhibition has been shown to mitigate airway inflammation, remodeling, and hyperresponsiveness induced by GPCR receptor production, including acetylcholine muscarinic receptors and histamine receptors, in airway smooth muscle [54]. In COPD, the production of GPCRs such as acetylcholine muscarinic, histamine, and protease-activated receptors can be stimulated by gastric acid, and importantly, GERD is caused by frequent or constant reflux of gastric acid, which would be compounded in subjects with COPD and GERD [25,27,29].

In addition to IL6/*STAT3* and GPCR signaling pathways, we also identified variants involved in oxidative stress mechanisms as potential modulators in both GERD and COPD. Among the top variants in our study, we identifed an association between variants within the *DPYD* gene and COPD only in multi-ancestry participants. *DPYD* encodes dihydropyrimidine dehydrogenase, which has been associated with chemosensitization, esophageal cancer, and NADPH oxidation [55,56]. Oxidative stress is an important mechanism in COPD pathogenesis and antioxidant medications, including NADPH oxidase inhibitors have been proposed as COPD therapeutics [56]. *DPYD* is targeted by eniluracil, which is currently on trial for oesophageal and digestive system cancers [57]. Interestingly, the top multi-ancestry loci associated with COPD only were significantly enriched for esophageal cancer and airflow obstruction. Oxidative stress induced by reflux of gastric acid is also an important mechanism in GERD and could further exacerbate COPD by serving as an additional pro-inflammatory factor [22]. Given that oxidative stress adversely affects both COPD and GERD, targeting *DPYD* via existing drugs could be a novel antioxidative treatment worthy of further investigation. We identified an ancestry-independent variant (rs78326497) within one locus that was associated with an increased risk of comorbid GERD and COPD in the multi-ancestry participants. This variant is intronic to *LINC02493*, which encodes long intergenic non-protein coding RNA (lncRNA) 2493. LncRNAs may play an important role in cellular responses to cigarette exposure and COPD pathogenesis [58]. Specifically, tobacco smoke exposure alters the expression and function of lncRNAs, which could lead to mitochondrial dysfunction and oxidative stress in COPD [58]. LncRNAs have also been implicated in the pathogenesis of Barrett’s esophagus (BE) and esophageal carcinoma, both of which, share chronic GERD as a risk factor [59]. Another locus associated with co-morbid GERD and COPD fell within *HCG17* (OR = 16.4), which encodes a lncRNA and is a known enhancer for *TRIM26* [60]. Both *HCG17* and *TRIM26* have been previously associated with COPD (in smokers and never smokers) and aerodigestive disorders [61,62]. *TRIM26*, whose closest neighbor is *HCG17*, encodes an E3 ubiquitin-protein ligase which plays a central role in determining response to oxidative stress by controlling levels of DNA glycosylases involved in the repair of damaged DNA [63,64].

Further, we identified a variant within the exonic region of *NID2* (rs147018937) associated co-morbid GERD and COPD in multi-ancestry participants. *NID2* encodes the Nidogen-2 protein, which plays a central role in basement member structure maintenance [65]. Oxidative stress has been shown to cause aberrations in basement member composition, affecting key pathological processes in both COPD and GERD [22,66]. Increasing our understanding of these molecules and their role in the pathogenesis of both COPD and GERD related complications could provide new insights into novel targets for COPD treatment.

Although our overall goal was to disentangle the common genetic etiology of COPD and GERD, we also assessed heritability and performed WGS analyses to identify genetic variants and regions associated with COPD and GERD alone. In doing so, we found the heritability of COPD in the combined AA participants to be 66.2% (h^2^ = 66.2%, 95% CI = 54.4–78.0), which is substantially higher than previous heritability estimates of COPD in AA participants (h^2^ = 37.9%, 95% CI = 17.5–58.3) obtained using genotype array data, however, it is important to note that the upper 95% estimate for the genotype array heritability estimate falls within the tail end of the 95% CI for heritability estimated using WGS data [11]. Smaller sample sizes have precluded rare variant estimates of heritability in AA participants in previous studies and by using WGS data, we may have accounted for missing heritability in AA participants, explaining why our estimates are substantially higher than previous studies [67]. We estimated the heritability of COPD only to be 28.3% in NHW, which is slightly lower than previous heritability estimates of COPD. This may be explained by our exclusion of participants with GERD from our COPD analyses. Given the high estimate for co-morbid GERD and COPD, it is plausible that a proportion of the heritability captured in previous estimates from studies that investigated only COPD may be attributable to overlapping biological pathways consistent with GERD.

Our study has many strengths but also limitations. We harnessed a large sample size of deeply phenotyped individuals with cutting-edge whole-genome sequencing to investigate the shared etiology of co-morbid GERD and COPD. We were able to implicate genetic loci associated with co-morbid GERD and COPD, which reflect known biology in GERD and COPD pathogenesis. Limitations of this study include the self-reported GERD phenotype. Sample sizes were large however, were not large enough to estimate heritability in all subsets of participants. Heritability analyses typically require many unrelated samples (>5,000), and due to these constraints, we were unable to calculate reliable heritability estimates for some subsets of participants particularly those of AA ancestry. As GERD can often present silently, particularly in COPD, it is possible that some controls may have had GERD, biasing our results towards the null. Likewise, our GWAS analyses may have been underpowered given that there are 82 known loci associated with COPD [68] from which only 5 loci (*FAM13A*, *CHRNA3*, *CHRNA5*, *RIN3*, and *HHIP*) were significantly associated with COPD in our analyses. Additional known COPD loci were suggestively associated (P<5E-06) likely due to the power limitation. Given this less stringent level, we acknowledge the caveat that we may have some false positives, which is why we termed them suggestive. We also employed a Bonferroni-corrected level of significance to gene-based findings which may have been overly conservative. Although we performed multi-ancestry and ancestry-stratified analyses, most of the participants included in this analysis were of NHW descent. This underscores the need for additional research to identify additional variants and regions associated with comorbid GERD and COPD that are shared across ancestries to aid in the development of personalized medicine interventions that are generalizable to all participants.

In conclusion, we have estimated the co-heritability of GERD and COPD and identified novel loci associated with comorbid GERD and COPD supporting a role for common etiology between both diseases. These loci may prove useful for insight into the development of preventative therapies as well as provide rationales for future mechanistic research into precision medicine targets for COPD via the inhibition of GERD. Future directions building upon this body of work will include Mendelian randomization analyses to investigate causality between COPD and GERD. Larger cohorts containing more ancestral diversity are needed to further elucidate the biological mechanisms of co-morbid GERD and COPD to hopefully uncover novel and increasingly generalizable insights into COPD pathogenesis.

## Methods

Institutional Review Board approval was obtained for the research from the University of Alabama at Birmingham (Approval Number: 300009289). All COPDGene, ECLIPSE, and SPIROMICS participants provided written, informed consent, to participate in each study and IRB approval was obtained at each of the clinical centers.

### Study participants

Details of the COPDGene, ECLIPSE, and SPIROMICS cohort studies have been previously described [69–70]. Briefly, COPDGene (NCT00608764, www.copdgene.org) is a multi-center longitudinal cohort study designed to examine the genetic risk factors for COPD [69]. Subjects were recruited between the ages of 45 and 80 with at least a 10 pack-year smoking history. Exclusion criteria included pregnancy, history of other lung diseases besides asthma, prior lobectomy or lung volume reduction surgery, active cancer undergoing treatment, or known or suspected lung cancer [69]. The SubPopulations and InteRmediate Outcome Measures in COPD Study (SPIROMICS) is a longitudinal, multicenter observational study of COPD designed to identify COPD subpopulations and to validate intermediate outcome measures in participants aged 41–80 with ≥20 pack-years at enrollment [68]. The Evaluation of COPD Longitudinally to Identify Predictive Surrogate Endpoints (ECLIPSE) study was a large, multicenter longitudinal observational study conducted over 3 years aimed at identifying clinically relevant COPD subtypes and novel biomarkers and genetic factors [70]. Participants aged 40–75 years with a smoking history of >10 pack-years were enrolled [70]. To maximize statistical power, multi-ancestry analyses included participants from all 3 cohorts irrespective of self-identified or genetic ancestry, combined NHW analyses including participants of NHW ancestry from all 3 cohorts, and combined AA analyses included participants of AA ancestry from COPDGene and SPIROMICS.

### Phenotype definitions

Phenotypes were defined at baseline enrollment for each cohort. COPD was defined using post-bronchodilator lung function testing (FEV_1_: forced expiratory volume in one second and FEV_1_/FVC: FEV1 expressed as a fraction of forced vital capacity < 0.7). In COPDGene and SPIROMICS, ascertainment of physician-diagnosed GERD was based on self-report [68,69]. The participant was presented with the question “Have you ever been told by a physician that you have GERD”. In ECLIPSE, GERD was defined at baseline as physician-reported reflux and/or heartburn [70]. Three models with a common set of controls (without COPD and without GERD) were used to disentangle the relationship between COPD and GERD: Model 1) Cases: Comorbid COPD and GERD and Controls; Model 2) Cases: COPD Only and controls; and Model 3) Cases: GERD only and controls.

### Genomic data and quality control

WGS data aligned to genome build GRCh38 was obtained from the Trans-Omics for Precision Medicine (TOPMed) program freeze 9. WGS data from the TOPMed program have undergone stringent quality control (QC), and harmonization and are available on the BioData Catalyst (BDC) platform [37] accessible with dbGaP approval (phs000951.v5.p5, phs001472.v2.p1, phs001927.v1.p1). All QC metrics described below for WGS data available on BDC were performed by TOPMed. On average, WGS data had deep (~30x) coverage with joint-sample variant calling and variant level QC [37]. Samples were removed if they exhibited unexpected relatedness, discrepancy between self-reported and genotype sex, or failed concordance between prior SNP genotypes and WGS-derived genotypes. Duplicate samples were identified using independent markers and only one subject from each pair was retained. Ancestral population groups were classified by confirming a match between self-reported and genetic ancestry. Participants whose self-reported ancestry differed from that estimated by genetics using principal component analysis were excluded from analyses. Variants were removed based on Mendelian discordance, a support vector machine (SVM), and an excess heterozygosity filter [37].

### Statistical analyses

**Fig 3:**
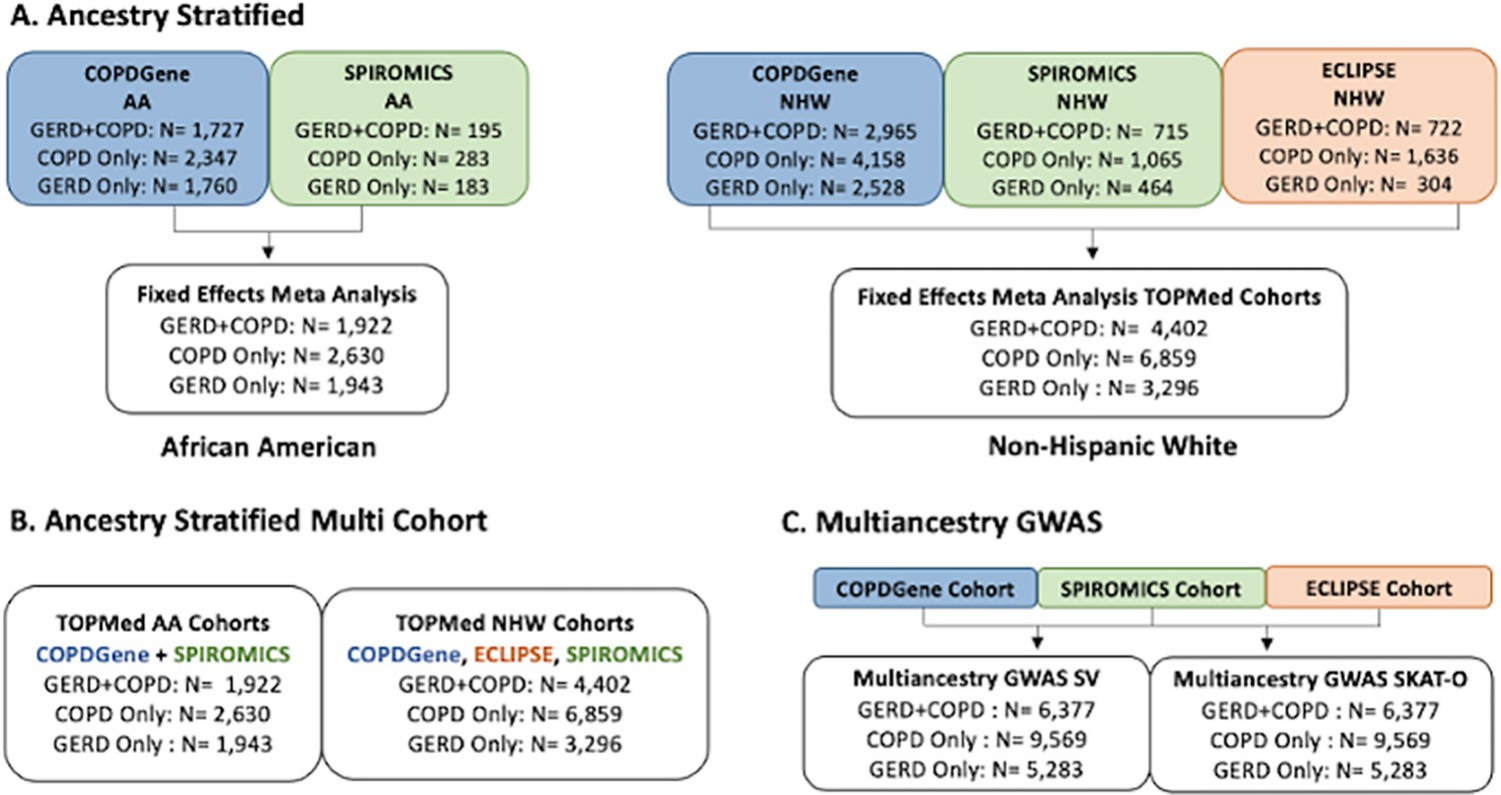
Study design for genome-wide association study (GWAS) analyses of whole-genome sequencing (WGS) data using the COPDGene (blue), SPIROMICS (green), and ECLIPSE TOPMed cohorts. A common set of controls with no COPD and no GERD were used for all 3 models.

Unless otherwise specified, statistical analyses were performed using R v3.6.0 or the BioData Catalyst platform [71]. Fig 3 provides a detailed overview of genomic analyses.

### Heritability and genetic correlation

Heritability was estimated utilizing individual-level WGS data in phenotype strata with 2,500 or more participants [co-morbid GERD and COPD, COPD only, and GERD only in combined NHW participants (COPDGene, ECLIPSE, and SPIROMICS), COPD only in combined AA participants (COPDGene and SPIROMICS), and COPD only in COPDGene alone] using the LDAK software [72]. We performed an additional heritability calculation of GERD, regardless of COPD status, in the combined NHW population. LDAK utilizes a generalized restricted maximum likelihood (REML) solver to estimate the heritability contributed by kinship matrices, which were calculated assuming the GCTA model for this analysis [73]. We adjusted for age, sex, pack-years of smoking, and the first 5 principal components of genetic ancestry. Prior to input into the LDAK software, the number of variants identified was pruned to a set-in linkage equilibrium (LE). The likelihood ratio test was used to test whether each estimate of heritability was different from zero. Significance was defined as p<0.05.

### GWAS analysis of TOPMed whole genome sequencing data

Each single nucleotide variant (SNV) with minor allele count (MAC) of 20 or greater was tested for its association with COPD only, GERD only, and comorbid COPD and GERD using an additive model adjusted for age, sex, pack-years, and the first 5 principal components to summarize genetic background using the SAIGE (Scalable and Accurate Implementation of Generalized mixed model) implemented using the GENESIS v1.1 pipeline [74,75]. SAIGE uses two main steps: 1) fitting a null logistic mixed model to estimate the variance component, and 2) testing for the association between each SNV and phenotypes by applying saddlepoint approximation (SPA) to the score test statistics to analyze large sample data while controlling for unbalanced case-control ratios and sample relatedness [75]. Regional association plots were generated using LocusZoom and linkage disequilibrium (LD) information from the 1000 Genomes African Ancestry and 1000 Genomes European Ancestry panels were used [76]. Genome-wide significance was defined as p<5.0E-08 and suggestive significance was defined as p<5E-06. Independent loci were characterized using Functional Mapping and Annotation of Genetic Associations (FUMA). FUMA functionally annotates GWAS summary statistic data and prioritizes the most likely causal SNVs and genes [77]. FUMA gene prioritization is based on a combination of positional mapping, expression quantitative trait loci (eQTL) mapping, and chromatin interaction mapping [77]. Loci identified ancestry independent if the statistical significance for lead variants increased in the multi-ancestry analyses indicating the association was not private to a single ancestry.

### Gene-based GWAS analysis of TOPMed whole genome sequencing data

The aggregate rare variant test was performed within the GENESIS mixed-model association testing framework for variants with a minor allele frequency (MAF) less than or equal to 5% and minor allele count (MAC) greater than 25. Coding variants were aggregated into gene-based groups using GENCODE v29 gene annotation [78]. Variants were further filtered using the coding filter 1 –stringent (C1-S) strategy to enrich for likely causal variants. The C1-S strategy, previously described in detail [79], included high confidence predicted loss of function variants, missense variants predicted to be deleterious, and in-frame indels or synonymous variants. The SKAT-O method was also used to perform rare variant collapsing and aggregation tests due to 1) its ability to combine the strengths of both the burden and SKAT test statistics; and 2) its optimized statistical power and robustness [80]. We adjusted for age, sex, pack years of smoking, and the first 5 principal components of genetic ancestry. Aggregate rare variant test significance was defined as p<2.5E-6 [81].

### Meta-analyses

Meta-analyses were performed using the METAL software in NHW (COPDGene, ECLIPSE, and SPIROMICS) and AA (COPDGene and SPIROMICS) participants separately and in multi-ancestry participants [82]. Meta-analyses were performed using single variant summary statistic data from each model (M1: COPD + GERD; M2: COPD only; M3: GERD only). Significant genetic loci were classified as ancestry-independent loci if both the METAL heterogeneity analysis test statistic was non-significant (p-heterogeneity < 0.1), and the meta-analysis p-value was smaller than the most significant single ancestry p-value.

### Fine mapping analysis

Fine mapping analyses were performed using Probabilistic Annotation INTegratOR (PAINTOR) to prioritize biological causality of SNVs obtained from WGS data [83]. PAINTOR implements a Bayesian approach that incorporates genetic association results, linkage disequilibrium (LD), and functional annotation to generate the posterior probability (PP) of causality for each variant. Fine-mapping regions were prioritized based on examination of regional association plots generated for each locus significantly associated with co-morbid GERD and COPD, COPD only, and GERD only in multiancestry participants using LocusZoom and by including variants ±500 kb from the lead variant. Single variant summary statistics were integrated with LD data by generating a matrix with pairwise Pearson correlations for each SNV, and a matrix functional annotation data for lung, esophageal, stomach, and duodenum tissue for SNVs overlapping with a given annotation. Functional annotations were used as prior probabilities and learned from the data via Empirical Bayes to prioritize causal variants. Candidate causal variants were estimated by posterior probability and observed association Z-scores.

### Pathway and tissue enrichment analysis

Gene-set enrichment analysis (GSEA) and tissue enrichment analyses were performed by inputting single variant summary statistics into the FUMA software to examine the known biology of single variants associated with comorbid GERD and COPD, COPD only, and GERD only from multi-ancestry, combined NHW, and combined AA participants [77]. Tissue enrichment was assessed using FUMA by testing whether collections of genes exhibit tissue-specified expression patterns based on the Genotype-Tissue Expression project version 8 data [84]. Significance was defined as q-value < 0.05. Featured pathways included statistically significant pathways that provided biological plausibility to our hypothesized pathogenesis mechanisms.

### Comparison of COPD and GERD genetic architecture to COPD only and GERD only

We delved into the overlap in variants between the combined COPD and GERD, COPD only, and GERD only GWAS analyses to confirm that the variants associated with the combined COPD and GERD phenotype are driven by associations in both the COPD only and GERD only GWAS’s. To find variants associated with co-morbid GERD and COPD driven by variants associated with both COPD only and GERD only, we performed an overlap analysis between variants suggestively associated (P< 5E-06) with co-morbid COPD and GERD with variants nominally associated (P< 0.05) with COPD only or GERD only in multi-ancestry, combined NHW, and combined AA GWAS analyses.

## Supporting information

S1 Table Descriptives of participants from TOPMed (COPDGene, SPIROMCS, and ECLIPSE) cohorts included in analyses. (XLSX)

S2 Table Independent significant loci (P<5E-06) associated with comorbid GERD and COPD from participants from COPDGene, ECLIPSE, and SPIROMICS. (XLSX)

S3 Table Genetic variants significantly associated (P<5E-06) with comorbid GERD and COPD in multiancestry participants from TOPMed cohorts (COPDGene, ECLIPSE, SPIROMICS) with WGS data. (XLSX)

S4 Table Genetic variants associated (P<5E-06) with comorbid GERD and COPD in combined NHW populations from TOPMed (COPDGene, ECLIPSE, SPIROMICS). (XLSX)

S5 Table Genetic variants significantly associated (P<5E-06) with comorbid GERD and COPD in combined AA populations from TOPMed (COPDGene and SPIROMICS). (XLSX)

S6 Table Top 5 regions from aggregate association testing of GERD and COPD. **Aggregate association testing was performed using SKAT-O software.** Significance was defined as P<2.5E-06.
(XLSX)

S7 Table Gene-set enrichment analysis of genes associated with comorbid GERD and COPD from multiancestry, combined NHW, and combined AA participants from TOPMed cohorts (COPDGene, ECLIPSE, and SPIROMICS). Abbreviations: COPD—Chronic Obstructive Pulmonary Disease; GERD—Gastroesophageal Reflux Disease; Q-Value—FDR corrected P-Value.
(XLSX)

S8 Table Independent significant loci (P<5E-06) associated with COPD from COPDGene, ECLIPSE, and SPIROMICS. (XLSX)

S9 Table Genetic variants significantly associated (P<5E-06) with COPD in multiancestry populations from TOPMed (COPDGene, ECLIPSE, SPIROMICS). Abbreviations: COPD—Chronic Obstructive Pulmonary Disease; LL—95% Confidence Interval Lower Limit; MAF—Minor Allele Frequency; OR—Odds Ratio.
(XLSX)

S10 Table Genetic variants significantly associated (P<5E-06) with COPD in combined NHW populations from TOPMed (COPDGene, ECLIPSE, SPIROMICS). Abbreviations: COPD—Chronic Obstructive Pulmonary Disease; LL—95% Confidence Interval Lower Limit; MAF—Minor Allele Frequency; NHW—Non-Hispanic White; OR—Odds Ratio.
(XLSX)

S11 Table Genetic variants significantly associated (P<5E-06) with COPD in combined AA populations from TOPMed (COPDGene and SPIROMICS). Abbreviations: COPD—Chronic Obstructive Pulmonary Disease; LL—95% Confidence Interval Lower Limit; MAF—Minor Allele Frequency; NHW—Non-Hispanic White; OR—Odds Ratio.
(XLSX)

S12 Table Top 5 regions from aggregate association testing of COPD Only. Aggregate association testing was performed using SKAT-O software. Significance was defined as P<2.5E-06.
(XLSX)

S13 Table Gene-set enrichment analysis of genes associated with COPD Only in multiancestry, combined NHW, and combined AA participants from TOPMed cohorts (COPDGene, ECLIPSE, and SPIROMICS). Abbreviations: COPD—Chronic Obstructive Pulmonary Disease; Q-Value—FDR corrected P-Value.
(XLSX)

S14 Table Independent significant loci (P<5E-06) associated with GERD from COPDGene, ECLIPSE, and SPIROMICS. (XLSX)

S15 Table Mega-analysis of genetic variants significantly associated (P<5E-06) with GERD in participants from TOPMed cohorts (COPDGene, ECLIPSE, SPIROMICS) with WGS data. GWASs were run in all participants without ancestry exclusion using GENESIS single variant association testing. Abbreviations: AA—African American; COPD—Chronic Obstructive Pulmonary Disease; LL—95% Confidence Interval Lower Limit; MAF—Minor Allele Frequency; OR—Odds Ratio.
(XLSX)

S16 Table Genetic variants significantly associated (P<5E-06) with GERD in combined NHW populations from TOPMed (COPDGene, ECLIPSE, SPIROMICS). Abbreviations: AA—African American; Alt—Alternative Allele; Chr—Chromosome; COPD—Chronic Obstructive Pulmonary Disease; Freq—Frequency; LL—95% Confidence Interval Lower Limit; MAF—Minor Allele Frequency; OR—Odds Ratio; Pos—Position; Ref—Reference Allele.
(XLSX)

S17 Table Genetic variants significantly associated (P<5E-06) with GERD in combined AA populations from TOPMed (COPDGene and SPIROMICS). Abbreviations: AA—African American; Alt—Alternative Allele; Chr—Chromosome; COPD—Chronic Obstructive Pulmonary Disease; Freq—Frequency; LL—95% Confidence Interval Lower Limit; MAF—Minor Allele Frequency; OR—Odds Ratio; Pos—Position; Ref—Reference Allele.
(XLSX)

S18 Table Top 5 regions from aggregate association testing of GERD Only. Aggregate association testing was performed using SKAT-O software. Significance was defined as P<2.5E-06.
(XLSX)

S19 Table Gene-set enrichment analysis of genes associated with GERD Only in multiancestry, combined NHW, and combined AA participants from TOPMed cohorts (COPDGene, ECLIPSE, and SPIROMICS). Abbreviations: AA—African American; NHW—Non-Hispanic White; Q-Value—False Discovery Rate (FDR) corrected p-value.
(XLSX)

S1 Text Results for COPD only and GERD only single variant, gene-based, meta-analysis, and pathways and tissue enrichment analyses. (DOCX)
