# Supplementary material for: Novel risk loci encompassing genes influencing STAT3, GPCR, and oxidative stress signaling are associated with co-morbid GERD and COPD
Source: PLoS Genet. 2025 Feb 7;21(2):e1011531. doi: 10.1371/journal.pgen.1011531 (PMC11805425; doi:10.1371/journal.pgen.1011531)
Supplement: S1 Text — (DOCX) [file pgen.1011531.s020.docx]

**Supplemental Material:**

**Novel Risk Loci Encompassing Genes Influencing *STAT3*,**

**GPCR, and Oxidative Stress Signaling are Associated**

**with Co-morbid GERD and COPD**

**Results**

*Novel and Known Single SNVs Associated with COPD*

A total of 821 variants comprising 130 independent loci were associated with COPD only in the multi-ancestry, combined NHW, and combined AA populations (S8-S11 Table). In multi-ancestry participants, 235 variants and 54 loci were associated with COPD (p< 5e-06) (Table 3 and S9 Table). Among the 235 variants, 5 variants were also associated with COPD only among multi-ancestry participants at a level of genome-wide significance (S9 Table). Among these were 4 variants (4:88945562, 4:88939679, 4:88948767, and 4:88951941) intronic to *FAM13A*, an established COPD risk loci [OR= 1.22, 95% CI= 1.18-1.26, P= 6.83E-09; OR= 1.22, 95% CI= 1.18-1.26, P= 8.71E-09; OR= 1.21, 95% CI= 1.17-1.25, P= 3.13E-08; OR= 1.21, 95% CI= 1.17-1.25, P= 4.13E-08, Table 3 and S9 Table]. We also identified one variant intronic to *CHRNA3* (15:78606381) significantly associated with COPD [OR= 1.23, 95% CI= 1.19-1.28, P=1.07E-08, Table 3 and S9 Table]. Variants within *HHIP*, *RIN3,* and *CHRNA5*, other known COPD loci, were only suggestively associated with COPD in multi-ancestry participants (S9 Table). In addition, we identified two variants within the exonic region of *CRAMP1* (16:1668109) and *CHRNA3* (15:78618839) suggestively associated with COPD in multi-ancestry participants [OR= 0.44, 95% CI= 0.37-0.52, P= 3.71E-07; OR= 1.19, 95% CI= 1.15-1.23, P= 8.30E-07, respectively, S9 Table]. In combined NHW participants, 410 variants comprising 32 independent loci were associated with COPD only (S8 and S10 Tables). The top variant associated with COPD only in combined NHW participants (14:92631994, OR= 0.75, 95% CI= 0.71-0.79, P=6.57E-08, S10 Table) is intronic to *RIN3*, a known COPD locus. Variants within two additional genes, *HHIP* and *FAM13A*, previously associated with COPD*)* were also suggestively associated with COPD in the combined NHW participants (S10 Table). In the combined AA participants, 176 variants within 44 independent loci were associated with COPD only (S8 and S11 Tables). One variant (11:121178427), intronic to *TECTA*, was significantly associated with COPD in the combined AA participants [OR= 1.52, 95% CI= 1.41-1.64, P=2.01E-08, S11 Table].

*Gene-based Regions of Rare Variants Associated with COPD Only*

In the multi-ancestry participants, gene-based analyses of rare variants indicated *JCHAIN* was the top gene region associated with COPD (transcript ENST00000510614, P= 1.28E-05, S12 Table) although it did not reach genome-wide significance. Among the combined NHW participants, rare variants within the *NKX1* gene were associated with COPD (P= 1.59E-05, S12 Table). In the combined AA participants, rare variant analyses revealed the most significant gene associated with COPD was *P4HB* (ENST00000331483, P= 1.04E-03, S12 Table).

*Gene-set Enrichment Analyses of Genes within COPD Associated Loci*

Among multi-ancestry participants, genes mapping to loci associated with COPD only were enriched with genes associated with post bronchodilator FEV1/FVC ratio (q-value= 0.00054, S13 Table), acetylcholine binding (REACTOME Acetylcholine Binding and Downstream Events, q-value= 0.027, S13 Table), among other known gene sets. Genes mapping to loci associated with COPD among NHW participants showed enrichment for genes associated with behavioral response to nicotine (GO Behavioral Response to Nicotine, q-value= 2.93E-07, S13 Table), genes with hypermethylated DNA in esophageal squamous cell carcinoma (Tanaka methylated in esophageal carcinoma, q-value= 6.08E-05, S13 Table), and current cigarettes per day in chronic obstructive pulmonary disease (q-value= 1.17E-04, Table S13), among others. No gene set within the combined AA participants withstood correction for multiple comparisons.

*Novel and Established Single SNVs Associated with GERD*

A total of 143 single variants comprising 77 independent loci were suggestively associated with GERD in the multi-ancestry, combined NHW, and combined AA populations (Tables S14-S17). In the multi-ancestry participants, 64 single variants within 33 independent loci were associated with GERD only (Tables 4 and S14). The top variant associated with GERD only among multi-ancestry participants was intronic to *SELPLG* [OR= 2.17, 95% CI= 1.88-2.49, P=7.77E-08, Table S15]. One variant within the exonic region of *RREB1* was suggestively associated [OR= 17.27, 95% CI= 10.10-29.53, P=4.55E-06, Table S15] with GERD in multi-ancestry participants (Table S15). In addition, five of the top loci associated with GERD in the multi-ancestry participants comprising the *PTMAP5*, ENSG00000258081 (*MIR4307HG*), *COL5A1*, *ST8SIA1*, *C2CD5*, and *ELMOD1* genes were ancestry independent (Table S14). In the combined NHW participants, 30 variants comprising 15 independent loci were associated with GERD only (Tables S14 and S16). The top variant associated with GERD only in the combined NHW participants was 13:82006238 [OR=2.80, 95% CI= 2.31-3.40, P=1.80E-07, Table S16]. In the combined AA participants, 49 single variants comprising 29 independent loci were associated with GERD only (Table S14 and S17). The top variant associated with GERD only in the combined AA participants (14:62821284) is intronic to *KCNH5* [OR= 2.24, 95% CI= 1.94-2.59, P=7.51E-08, Table S17]. One missense variant within the exonic region of *SLC26A8* was suggestively associated with GERD only [OR= 10.65, 95% CI= 6.72-16.88, P=4.63E-06, Table S17] in the combined AA participants.

*Gene-based Regions Associated with GERD Only*

Among multi-ancestry participants, gene-based analyses of rare variants indicated *SLC26A8* was significantly associated with GERD only (ENST00000394602, P= 2.46E-06, Table S18). Among the combined NHW participants, gene-based analyses of rare variants identified *UGGT2* as the top gene associated with COPD only (ENST00000376747, P= 8.62E-05, Table S18). In the combined AA participants, rare variant analyses revealed three splice transcript variants (ENST00000355574, ENST00000490799, and ENST00000394602) mapping *SLC26A8* gene were significantly associated with GERD (Table S18).

*Gene-set Enrichment Analyses of Genes Within GERD Associated Loci*

Genes mapping to loci associated with GERD among multi-ancestry participants were enriched with genes within the ensemble cytogenic band chr6p21 (q-value= 4.68E-06, Table S19) and chr5q11 cytogenic band (q-value= 3.26E-05, Table S19). Genes mapping to loci associated with GERD among AA participants showed enrichment for involvement in the response to oxidative stress (Weigel Oxidative Stress Response, q-value= 0.011, Table S19), among others. No gene set within the combined NHW participants withstood correction for multiple comparisons.
